# WGDTree: a phylogenetic software tool to examine conditional probabilities of retention following whole genome duplication events

**DOI:** 10.1186/s12859-022-05042-w

**Published:** 2022-11-24

**Authors:** C. Nicholas Henry, Kathryn Piper, Amanda E. Wilson, John L. Miraszek, Claire S. Probst, Yuying Rong, David A. Liberles

**Affiliations:** 1 Department of Biology and Center for Computational Genetics and Genomics, Temple University, Philadelphia, PA 19122 USA; 2 Department of Biology, Haverford College, Haverford, PA 19041 USA; 3 Present Address: Department of Biological Sciences, University at Albany, Albany, NY 12222 USA; 4 Present Address: Genetics Area Program, University of Missouri, Columbia, MO 65211 USA; 5 Present Address: Groningen Institute for Evolutionary Life Sciences, University of Groningen, 9747 AG Groningen, The Netherlands

**Keywords:** Whole genome duplication, Phylogenetic analysis, Gene duplicability, Mutational opportunity

## Abstract

**Background:**

Multiple processes impact the probability of retention of individual genes following whole genome duplication (WGD) events. In analyzing two consecutive whole genome duplication events that occurred in the lineage leading to Atlantic salmon, a new phylogenetic statistical analysis was developed to examine the contingency of retention in one event based upon retention in a previous event. This analysis is intended to evaluate mechanisms of duplicate gene retention and to provide software to generate the test statistic for any genome with pairs of WGDs in its history.

**Results:**

Here a software package written in Python, ‘WGDTree’ for the analysis of duplicate gene retention following whole genome duplication events is presented. Using gene tree-species tree reconciliation to label gene duplicate nodes and differentiate between WGD and SSD duplicates, the tool calculates a statistic based upon the conditional probability of a gene duplicate being retained after a second whole genome duplication dependent upon the retention status after the first event. The package also contains methods for the simulation of gene trees with WGD events. After running simulations, the accuracy of the placement of events has been determined to be high. The conditional probability statistic has been calculated for *Phalaenopsis equestris* on a monocot species tree with a pair of consecutive WGD events on its lineage, showing the applicability of the method.

**Conclusions:**

A new software tool has been created for the analysis of duplicate genes in examination of retention mechanisms. The software tool has been made available on the Python package index and the source code can be found on GitHub here: https://github.com/cnickh/wgdtree.

**Supplementary Information:**

The online version contains supplementary material available at 10.1186/s12859-022-05042-w.

## Background

Gene duplication is an important driver of the evolution of genomes because without gene duplication, evolution is thought to act conservatively [1]. Gene duplication can relax selective constraints and enable faster evolution by creating redundant copies [2]. This redundancy can provide opportunity for favorable morphological innovative development in addition to other processes [3, 4]. Duplicate gene evolution comes in two broad types, smaller scale duplication (SSD) and whole genome duplication (WGD) which is rarer but in some cases can be a beneficial process [5]. These are differentiated by several functional features. Because all or a large piece of the genome is duplicated, WGD duplicates are duplicated together with their physical interacting partners [6–8].

Dosage balance theory states that selection favors gene products existing in stoichiometric balance which prevents the deleterious interaction of imbalanced partners [9–11]. Whole genome duplication (WGD) events preserve the dosage balance, so selection favors a slow initial duplicate gene loss rate [8, 12–17]. Alternatively, dosage constraints can favor removing gene duplicates quickly after small-scale duplication events when these events immediately throw off the stoichiometric balance of gene products [18]. Changes in gene expression can sometimes also aid in the initial retention of duplicate genes because it helps maintain the balance [14, 19]. Other processes that lead to the long-term retention of gene duplicates include subfunctionalization and neofunctionalization [1, 12, 20–22].

Duplicate genes that are retained over long evolutionary time periods do show patterns consistent with neofunctionalization and/or subfunctionalization [23]. Several factors affect the probability that an individual gene will neofunctionalize or subfunctionalize, including the number and specific functions of the gene, its length, and the complexity of its regulatory regions, among others [24–26]. The gene duplicability hypothesis states that some genes are more duplicable than other genes because of these gene characteristics [24, 27]. A naive expectation from this hypothesis is that when a genome undergoes consecutive WGD events, the genes retained after the first event are also more likely to be retained in following WGD events.

In a study performed on gene retention rates after consecutive whole genome duplication events in Atlantic salmon, a new statistic was developed [23]. This new statistic was developed for evaluating the conditional probability of duplicate gene retention from the second WGD based upon the retention status from the first WGD event for the analysis of the Atlantic salmon genome, which has had two relatively recent WGD events in its history [23]. The statistic is applied to a set of gene trees with consecutive WGD events on different species tree lineages. The probability ratio statistic is shown in Eq. 1.

1 $$Pratio= \frac{P\left(retained \,after \,WGD\, \#2 \right| retained \,after \,WGD \#1)}{P\left(retained \,after \,WGD\, \#2 \right| \,not \,retained\, after \,WGD \,\#1)}$$

The analysis from the Atlantic salmon genome unexpectedly gave a ratio of ~ 1, consistent with independence of duplicate retention in genes between events rather than the prior conceptualization of gene duplicability. Other lines of evidence might still support a more complex process involving non-independence [23, 28]. Because of this result there is interest in generalized software to characterize more genomes to enable analysis of the ratio and mechanisms leading _t_o retention generally and in different genomes. This type of analysis has the potential to spawn advancements in our understanding of duplicate gene retention together with additional future methodological refinement. Further, modeling with the gene duplicability hypothesis under different evolutionary scenarios and duplication times represents a parallel research track to explain the result from the Atlantic salmon genome. Other hypotheses beyond gene duplicability, including changes to mutational opportunity for functional change after duplicate gene retention can also be conceived and are being developed. It is important to note that the retention process is time dependent, meaning that the time between the WGD events and the time since the most recent event affects the probability that duplicate copies are retained. Evaluating this hypothesis requires modeling that is time-dependent and will be presented elsewhere.

To generate data across the genome from consecutive duplication events, a phylogenetic approach was developed [23, 28]. This approach relied upon the construction of gene trees for all genes in the genome and a reference species tree, with the need to differentiate between smaller scale events and the whole genome events of interest. The original script [28], which was based upon algorithms for gene tree/species tree reconciliation, was hard coded for properties of salmonids, including syntenic information in the Atlantic salmon [23] and rainbow trout [29] genomes.

Other phylogenetic methods of identifying and differentiating SSDs and WGDs rely on the number of gene trees that show the event in question [30, 31]. Additionally, some methods to strengthen the identification of WGD events can use syntenic information because one expects conserved synteny after a WGD event but not with SSD events [32]. This information provides support for the identified events and complements a purely phylogenetic approach.

Here, using the Python scripting language, generalized software to calculate the probability ratio for any pair of WGD events in a tree specified by a user has been created. The software takes a collection of gene trees and a species tree as input in doing so and is available at https://github.com/cnickh/wgdtree.

## Implementation

### Algorithm for inference

Software has been created to enable evaluation of conditional retention probability ratios (the test statistic) for any genome that has a pair of WGD events in its history. Written in Python, the conditional probability ratio statistic is calculated from a collection of gene trees derived from systematic comparative genomic analysis and a reference species tree with whole genome duplication events labeled. The statistic can be calculated for every pair of WGD events that occurs serially in the evolutionary history of a species. This refers to two whole genome duplication events that occurred on different species tree lineages that are both on the same phylogenetic trajectory from the species tree root to the extant species tip. At the heart of the package, is a gene tree-species tree algorithm that labels specific nodes in each gene tree, as described below.

The input to the software for inference are a reference rooted species tree and a set of unrooted gene trees from the genomes of the species involved. Gene trees do not need to contain genes from all species in the species tree but are assumed to contain all members that existed descended from the root node of the species tree. Once rooted using the species tree, gene trees containing more than 1 species with root nodes that are duplication events are split iteratively until the root node is a speciation event.

In order to calculate the conditional probability ratio, it is needed to map WGD events onto nodes of a gene tree. In principle, a WGD event could correspond to any duplication event on a gene tree where all leaves under that node correspond to a species under the event on the species tree. Here it will be assumed the most parsimonious solution, where the placement that results in the smallest number of small scale duplications is utilized. When equally parsimonious solutions are possible, the algorithm will place WGD at the earlier node. There may be scenarios where this choice can lead to a bias, but this has not been detected here. The mapping has a linear time complexity with respect to the number of nodes on the species tree.

### Notation

- **G**, denotes an input node. This should be the root node of a rooted and labeled gene tree.
- **S**, denotes an input node. This should be the root of the corresponding species tree with labeled WGD events.
- ***g***, is used to represent specified nodes on the gene tree.
- **s,** is used to represent specified nodes on the species tree.
- **r(n)**, denotes the right child of node **n**.
- **l(n)**, denotes the left child of node **n**.
- **L(n),** denotes all leaves under **n**.
- add_event(n,*), labels a gene tree node **n** as a WGD duplication, where the duplicate was either retained so the event is “Present” or the duplicate was lost so the event is “Missing”.
- **lca(s,g)**, denotes a method that returns the mapping of node ***s*** onto ***g*** by getting the last [least] common ancestor of all leaves present under ***s*** on ***g***. For example, let **g** define a gene tree node: **((a1, b1), (a2, b2))g**. Let **s** be a parent node on the species tree with only **a**,**b** as children. Here the left child and the right child include all species labels. Then **lca(s,g)** will simply return **g**.

### Implementation

The placement algorithm takes as input binary gene trees that are rooted and reconciled such that duplication and loss events are present and labeled and one species tree where the branches containing the WGD events are labeled. This is generated by the software as described below, given a rooted species tree and a set of unrooted gene trees. Given the root node of a gene tree G and the root node of the species tree S the algorithm maps the WGD events on to the gene tree to give as output a new gene tree with nodes labeled as WGD.

First it is necessary to root and reconcile the gene trees. The software package presented here implements a known reconciliation algorithm [33], which differs from an algorithm previously implemented in the group [34]. The software also roots the tree by iterating over all branches and selecting the root that minimizes the number of duplication and loss events on the reconciled tree. From the reconciliation a rooted gene tree with both duplication and loss events labeled is obtained. Although it is possible and computationally more efficient to map WGD events onto the gene tree as part of the reconciliation it was decided to keep these methods separate to allow other reconciliation algorithms to be used with the WGD placement software. As long as the resulting gene tree is labeled with duplication and loss events the placement algorithm will work. The loss events are treated as leaves for the purposes of the mapping. Let **G** be the root node of a gene tree and **S** the root of a species tree. Both should be labeled rooted binary trees. The set of leaves of a gene tree **L(G)** and a species tree **L(S)** are taken from the same set of species. The first step is to select the most recent possible node **n** under **g** an arbitrary gene tree node such that **L(s) ⊆ L(n)**, where **s** represents a node on the species tree directly after the WGD event and **L(s)** denotes all leaves under **s**. Let **lca(s, g)** be a function for this mapping. This mapping could return an **n** such that **L(n)** ⊈ **L(s**), for example if **g** was a node **(((a1, b1), c1), ((a2, b2), c2)))g** and **s** was only a parent to **a,b**.If this is the case **n** is a duplication occurring before the whole genome duplication event. This means it is possible the event corresponds to two locations on the gene tree. So we use **r(n)**, the right child of **n** and **l(n)**, the left child of **n** and get two new mappings. This process continues until we have a likely candidate, a node or nodes **n** such that **L(s) ⊆ L(n)** and **L(n) ⊆ L(s)**.

Next, one checks if **n** is labeled as a duplication by the rooting/reconciliation method. If the node is not a duplication, then the node is labeled as a missing WGD event. If the node was a duplication both children are checked for duplication events. If both children are duplication nodes, then the event is placed on both children. If not, the event is placed on the current node and labeled as present. The reasoning behind checking the children for duplication events works as follows. If a duplication, where all leaves under that node correspond to a species under the event on the species tree, is in fact not a result of WGD it be would expected to see both copies of the gene duplicate as a result of WGD. This method greatly reduces the number of duplication events attributed to small scale duplication compared to placement methods that do not check children node for duplications. Here, **event_num** is an integer that tracks which event is being placed. Upon successfully placing an event, it is incremented for the next consecutive event. For example, when **place_event()** is called if **event_num** is 0 the node will be labeled “event 0”.

### Pseudo-code

**Fig. 1:**
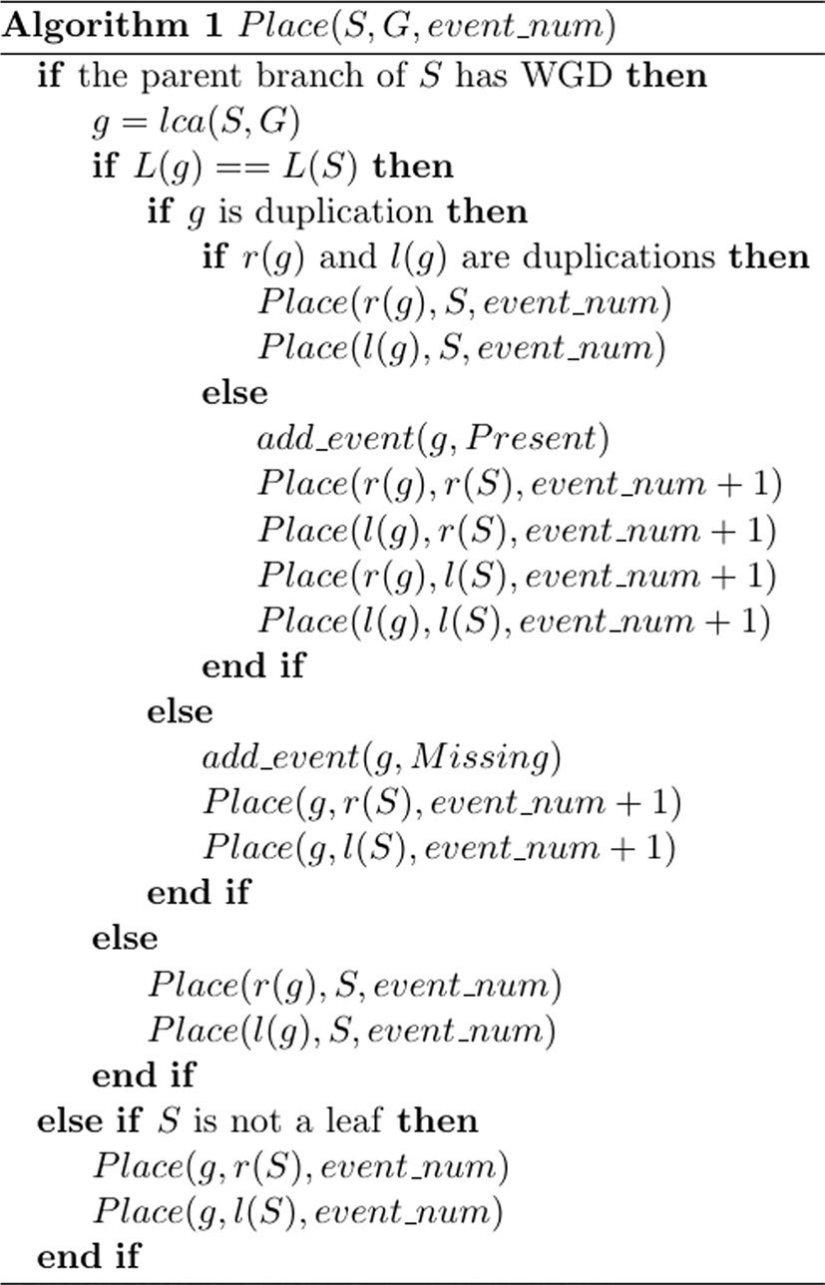
Pseudo-code for the algorithm is given in this figure

Pseudo-code for the algorithm is provided as Fig. 1.

Now with WGD events mapped onto the gene tree, computing the conditional retention is straight forward. First one iterates over each node until we find a node labeled as event 0 then we iterate over all descendant nodes until we find a node labeled as event 1. For every event 1 node found the number of possible duplicate copies increases by one. All the leaves descending from the event 1 node are checked and the total number of retained duplicate copies is counted. The number of duplicate copies along with the number of possible duplicate copies is added to a counter that tracks the total copies and possible copies across all gene families being analyzed. If event 0 was present, then the copies and possible copies get added to the RR count and if event 0 was missing, the copies get added to the LR count. This process repeats for all events 1 descending from the event 0. This is done for every event 0 node on the tree.

For example, In the case where a gene "A" was retained after the first WGD, there will be two copies of "A", "A1" and "A2", before the second WGD happens. If, after the second WGD, the new copies of "A1" are retained but not those "A2". The software with find one event 0 with two descended nodes labeled event 1 so 2 possible duplicate copies, and since the “A1” duplicate was retained and not “A2” we would see 1 duplicate copy of the gene still present on the tree. Thus for just this tree the conditional probability of RR would be 0.5 and since the first event was retained there would be no data for LR.

### Comparative genomic bioinformatic pipeline

The following seven species of plants with 5 pairs of WGD events between them [35–38] were selected to be analyzed: *Ananas comosus, Elaeis guineensis*, *Nelumbo nucifera*, *Oryza brachyantha*, *Panicum hallii, Phalaenopsis equestris*, and *Phoenix dactylifera*. All plant species selected are autopolyploids with available high-quality genomes, allopolyploids were eliminated. A species tree was generated using NCBI [39] with the timing of species divergences generated using Timetree [40]. Protein sequences for all seven species were gathered from NCBI as FASTA files. A total of 255,312 protein sequences were gathered, 35,775 from *Ananas comosus* at 400x, 41,887 from *Elaeis guineensis* at 16 × coverage, 38,191 from *Nelumbo nucifera* at 100 × coverage, 26,803 from *Oryza brachyantha* at 104x, 44,192 from *Panicum hallii* at 202 × coverage, 29,894 from *Phalaenopsis equestris* at 99.5 × coverage, and 38,570 from *Phoenix dactylifera* at 139 × coverage. High coverage genomes were used to reduce any potential for bias due to missing gene duplicates. It should be noted that the Pratio statistic is expected to be more robust than other retention statistics because missing duplicates might be expected to occur in the statistic numerator or denominator without bias. BLAST all-against-all was run for each pair of species, including against themselves, at an e-value threshold of 10^–10^ to identify homologous protein pairs. These pairs of homologs were run through a script to ensure that a pair of homologs had both percent identity and percent ungapped were both ≥ 60%. 255,187 gene families were formed by single linkage clustering of all the gene pairs. Protein alignments of the gene families were generated using MAFFT [41]. Maximum likelihood trees were created for gene families of size 4 or greater by PhyML [42] using SMS model selection [43] and Neighbor-Joining. Due to the size of some gene families (size > 100) PhyML was not an efficient method to use, Neighbor-Joining was used for these families. The gene trees were rooted using a Python script based off [7, 34]. As described above, gene trees with more than 1 species and a root node as a duplication event were iteratively split until the root node was a speciation event. In total 12,852 trees with size > 4 were generated, 81 of which used neighbor joining due to size.

### Generation of simulated data

For testing and as a companion to the inference tool, a simulation tool was built that is capable of producing a statistically probable gene trees for any given species tree [44–46]. Using a Poisson process to dictate the arrival of events (duplication/loss), a set of gene trees is produced. The evolutionary history of a gene is simulated over each branch of a given species tree.

Duplication affects the gene tree by duplicating the corresponding branch and subtree and losses affect the tree by dropping one branch and subtree. The simulation also includes the ability to add WGD duplication events where every copy of the gene present for the event is duplicated. The location for these events is determined via comments with a specific T value on the input species tree. The T value represents time and affects the probability that the simulation will place a SSD before or after the event on the same branch. This is a comment used with NHX format. The package uses ETE 3 [47] to read and manipulate trees. The resulting tree is a rooted simulated gene tree corresponding to the inputted species tree with WGD events labeled. Using these simulated trees, the inference method used to place WGD events on the tree can be tested for accuracy under different sets of conditions.

**Fig. 2:**
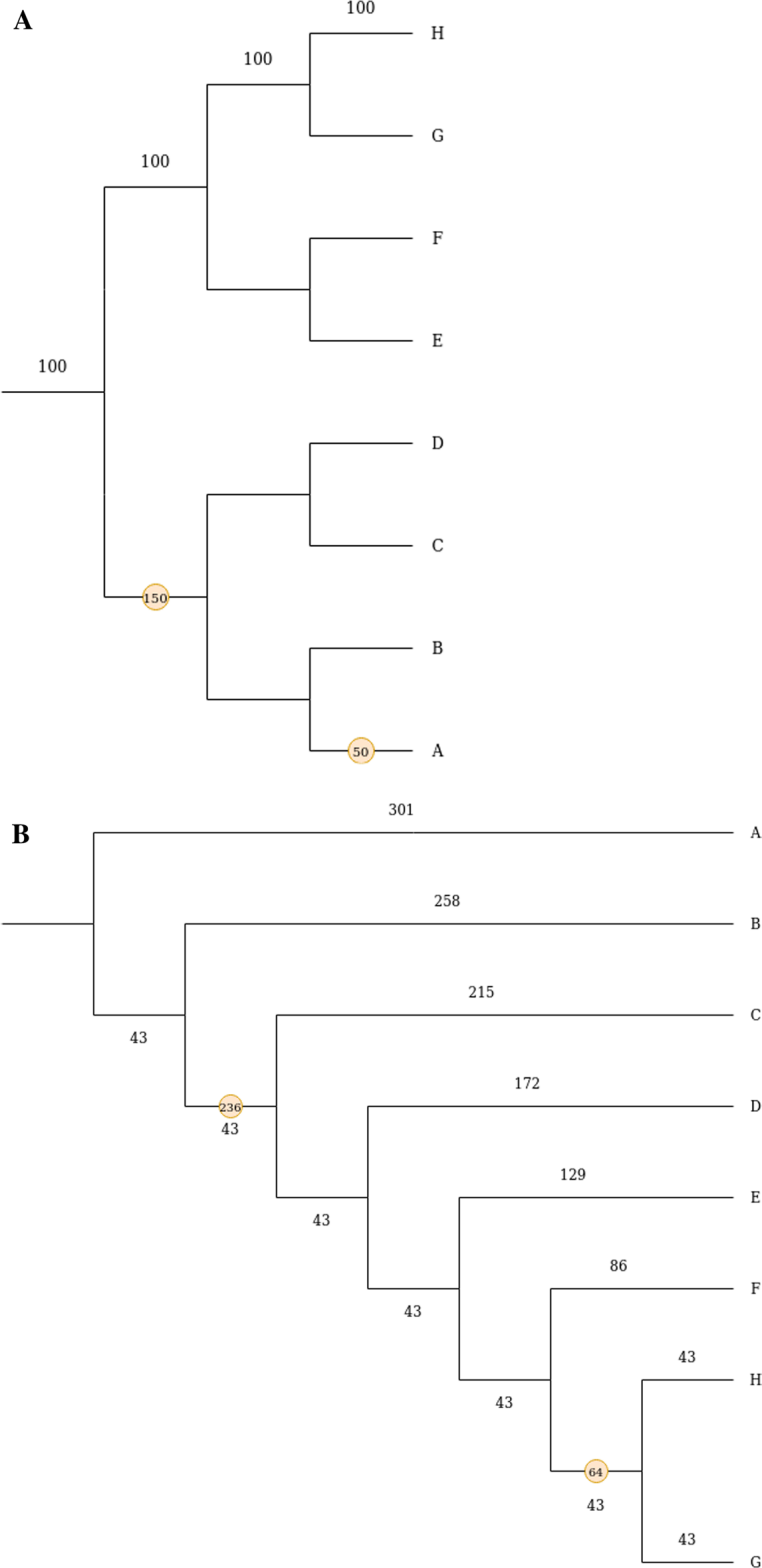
The trees in this figure represent the two tree types used to generate simulated phylogenetic trees in order to test the accuracy of the inference method. Branch lengths are in millions of year. The dots represent WGD events. The balanced (**A**) and caterpillar (**B**) trees shown here have events one and three speciation events apart respectively

The simulation method was run over two tree types, balanced and caterpillar (Fig. 2) to produce gene trees with correctly labeled WGD nodes. 1,000 trees were produced for every combination of loss and SSD rates = {0.01, 0.009, 0.002, 0.0002, 0.00002} (units of ‘event per million years’) (Additional file 1: Table S1). The values were chosen based on the current estimate of the rate of evolutionary events given in [48] and simulation run time limitations.

To test if the number of speciation events between WGD events affected the accuracy of the placement, the simulation was run for variations of the balanced and caterpillar tree where WGD events were placed a different number of speciation events apart. 1000 trees for each placement, loss rate and SSD rate were produced (Additional file 1: Tables S2 and S3). When the inference tool was run over the simulated trees to identify the branches inferred to have duplication events, the accuracy was inferred. Accuracy is the total number of correctly placed events divided by the total number of events called on the tree.

### Bootstrap analysis

Bootstrap analysis was performed to generate p-values and intervals containing 95% of the data. For the comparative genomic analysis, 1000 bootstrap samples of the 8013 trees containing *P. equestris* were generated. From the simulated data, 1000 bootstrap samples of the 1000 trees for each of the 150 data points was generated.

### Software user information

The tool developed in this paper is available on the Python package index under the name WGDTree from https://github.com/cnickh/wgdtree. The software provides functions for simulating likely gene tree phylogeny for a given species tree with WGD events, placing WGD events onto gene trees given a labeled species tree, and determining the conditional retention rate of duplicates resulting from WGD events. There is a user guide available with the source code on GitHub. In addition to the user guide there is also example code displaying the expected usage of the functions provided by this package.

## Results

Here, WGDTree is evaluated with simulated data to characterize its performance before being run on a plant genome taken from a larger comparative genomic study. The results of these analyses are shown below in presenting the new software.

### Simulated data

**Fig. 3:**
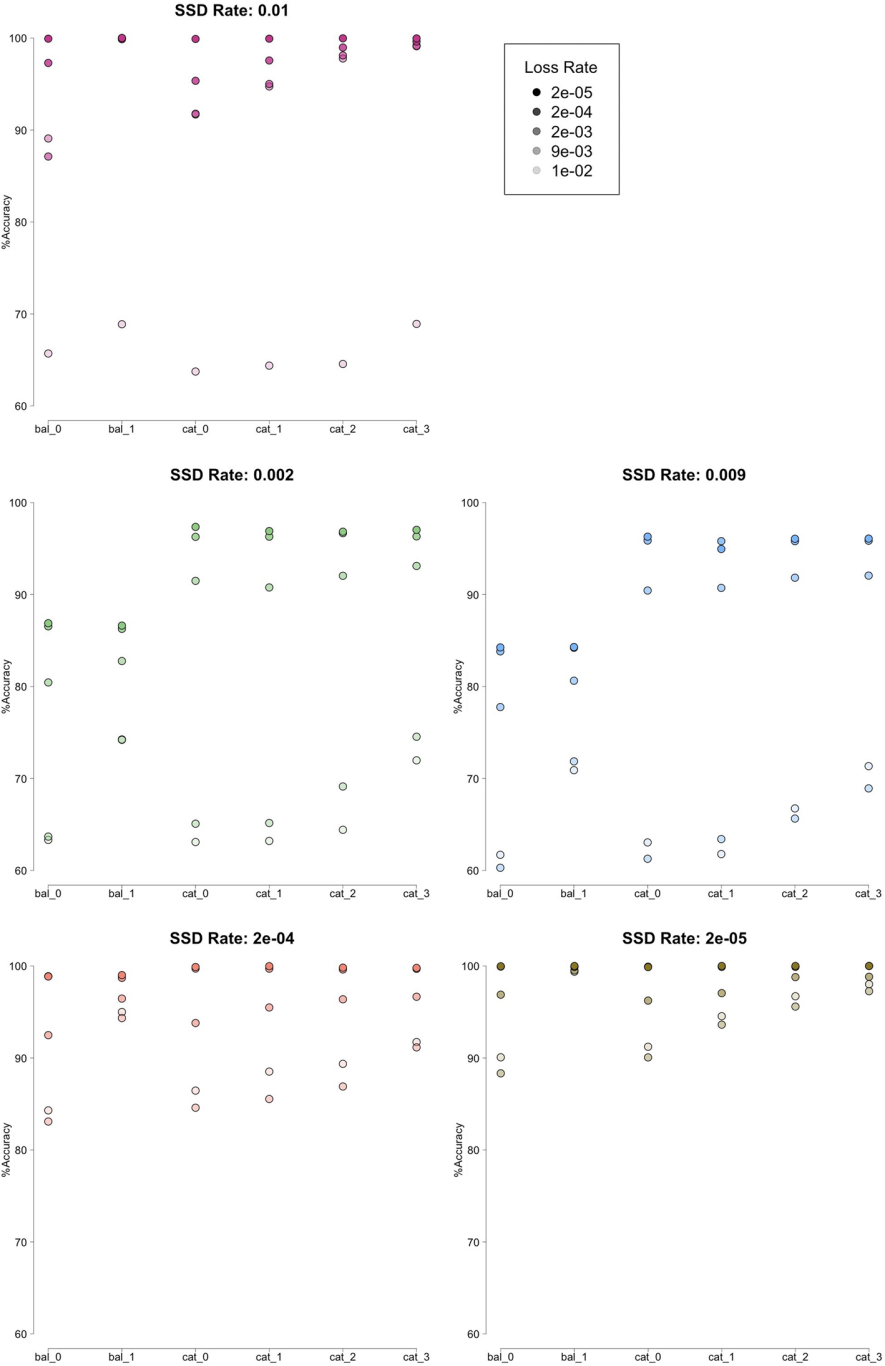
Each point shows the total accuracy (z) of the inference method’s placement of events across all simulated trees for a given species tree, loss (x) and SSD (y) rate in units per million years. The simulation conditions for each data point as described are where "bal_x" and "cat_x" represent a balanced and caterpillar tree type respectively, with WGD events placed "x + 1" speciation events apart on the tree

Using the set of simulated data, the accuracy for each of the trees of different tree topologies, SSD rates and loss rates was determined and is shown in Fig. 3 and is generally high. The tool’s accuracy on both caterpillar and balanced trees was higher with small loss rates, regardless of SSD rates, but did even better when the SSD rate was also small. For caterpillar trees, the accuracy increased as the distance (in branch length, also corresponding to the number of intervening speciation events) between the WGD events increased. The balanced tree type allowed for less distance between events making the overall accuracy lower. When distance between the events was the same, the tool performed better on the balanced tree type than on the caterpillar (equivalent data points had non-overlapping 95% confidence intervals in comparisons). With events spaced one speciation apart and high SSD and loss rates (0.01–0.002) accuracy was significantly higher on the balanced tree type (Additional file 1: Tables) (again, equivalent data points had non-overlapping 95% confidence intervals in comparisons).

The four distinct clusters in Fig. 3 are due to using noncontinuous loss and SSD rate parameters for the simulation. The four clusters are correlated with trees generated with; high (0.009, 0.01) loss and ssd rates, low(0–0.002) loss and SSD rates, high loss and low SSD rates, low loss and high SSD rates. The color of the data points indicates tree type.

### Application of the inference method to comparative genomic data

**Fig. 4:**
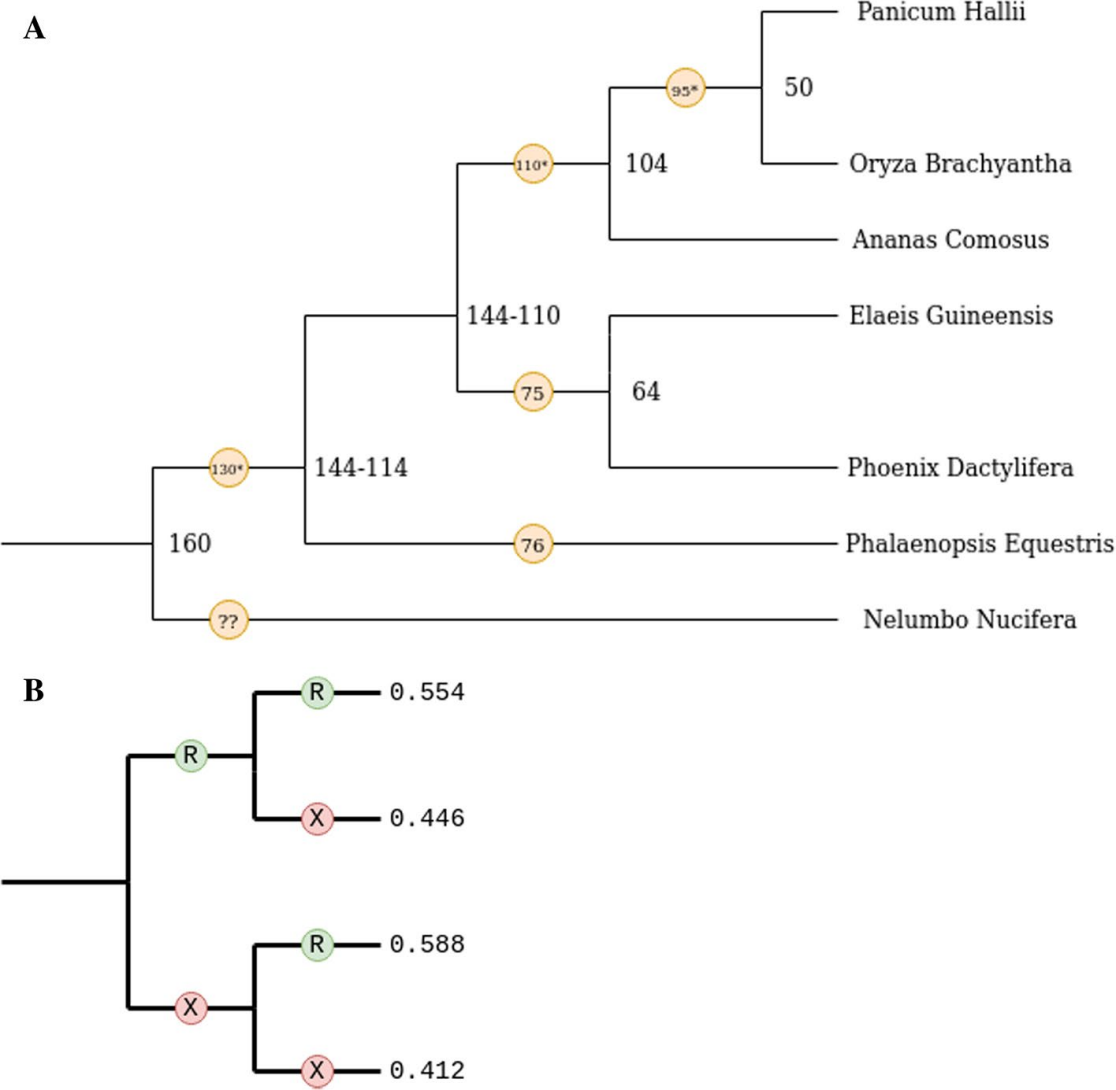
*P. equestris* Retention Rates. **A** This is the monocot species tree, where all units are in MYA. The events being analyzed are the events at ~ 130 MYA and 76 MYA. **B** From data analysis using WGDTree, *P. equestris* was found to have a Pratio of 0.94 (0.93–0.96 95% confidence interval)

To demonstrate the utility of the software tool presented here, one pair of recent whole genome duplication events on the lineage of *P. equestris* was analyzed (Fig. 4). The data was taken from a larger comparative genomic study that will be published elsewhere. Gene trees were created using the described methods. The software tool rooted the trees and calculated the conditional probability of retention of duplicates resulting from WGD. The conditional probability ratio (Pratio) was found to be 0.94, indicating that the calculated statistic is significantly smaller than the Pratio 1 (*p* < 0.001, the limits of the bootstrapping analysis that was performed). Results indicated that genes retained after the first event were not more likely to be retained again after the second event. Values for other genomes in the comparative study will be reported elsewhere as part of a paper focused on the underlying biology.

## Discussion

The Pratio for *P. equestris* from two recent WGD events ~ 130 million years ago (MYA) and 76 MYA was found to be 0.94. This finding is superficially similar to the Pratio found for Atlantic Salmon in previous work, which was ~ 1. Again, these results support that genes retained after the first event were not more likely to be retained again after the second event. Because the time since the most recent events are similar and the time between the WGD events was significantly shorter than the pair of WGD events explored in Atlantic Salmon, this result is particularly interesting because it potentially could have supported the hypothesis that the probability of retention is independent after consecutive events. In *P. equestris*, the ratio was statistically less than 1, which is not consistent with expectations of independence of retention between the two events. Future work needs to be done to identify more Pratio data points in other species with other WGD events in their lineages to determine if these results are consistent for WGD events of different ages.

The inference tool performed well for a range of tree topologies and SSD rates particularly when loss and small-scale duplication rates were small and when event pairs were placed further apart. Therefore, this software can be used to reliably calculate Pratio values in other lineages. Future modeling studies can use the data generated by this tool to identify the dominant process(es) involved in the retention of duplicate genes. Studying consecutive WGD events provide a unique opportunity to explore the dominant processes involved in the retention of duplicates because they can illuminate the probability each gene will remain as a duplicate after different lengths of time, and after its already been duplicated or lost. The results found in Atlantic Salmon and now *P. equestris* potentially call into question the gene duplicability hypothesis because they do not initially appear to align with the idea that some genes are more duplicable than other genes. Additional modeling is necessary to fully explore the expectations of the gene duplicability hypothesis. If the gene duplicability hypothesis is not supported through model testing, then this could challenge the extent to which the function and complexity of a gene’s interactions affect retention of the gene. However, the gene duplicability hypothesis could be supported if the hypothesis also interacts with the law of diminishing returns (either mutational opportunity for different events or reduced selection for events that are mutationally acessible), or even dosage constraints. Dosage balance may play a role in duplicate gene retention, especially in WGD events that are closer together and more recent. Alternatively, the hypothesis that the landscape of mutational opportunity could affect the likelihood of being subfunctionalized or neofunctionalized, affecting the probability of being retained as a duplicate, is a novel hypothesis in relation to gene duplication. Future work will model these hypotheses and conduct model testing on the data generated by the tool presented in this paper and identify what process(es) and to what extent do they affect the probability a gene will be retained as a duplicate. All of this analysis is supported by the software package described here, that produces processed Pratio data for analysis.

One caveat to this method is that homologs that are massively diverged are difficult to identify. Syntenic information would be helpful to incorporate in the algorithm because it would help identify orthologs and paralogs from WGD events [49]. A potential extension to this method could include analysis of syntenic regions in different genomes from genome alignments as a generalized feature, as was performed in the analysis of the Atlantic salmon genome [23].

## Conclusions

Here, we presented a useful software tool that is capable of rooting gene trees and reconciling them with species trees and then accurately identifying and differentiating between speciation events, WGD events, SSD events, and loss events. From there, it calculates this statistic developed for evaluating the conditional probability of duplicate gene retention from the second WGD based upon the retention status from the first WGD event. With this tool, the conditional probability ratio for *P. equestris* was determined to be 0.94, which like the conditional probability ratio of Atlantic Salmon does not result in a ratio greater than 1. More species that have undergone two recent duplication events can be identified to provide a large enough dataset for model testing.

### Availability and requirements

- Project name: WGDTree
- Project home page: https://github.com/cnickh/wgdtree. This package includes both the analysis tool and the tool for simulating the data that was used to test the analysis software.
- Operating system(s): Platform independent
- Programming language: Python
- Other requirements: 3.9.2 Python version
- License: GNU General Public License (GNU GPL)
- Any restrictions to use by non-academics: none

## Supplementary Information

**Additional file 1**. This file contains 6 supplemental tables that present the analysis of simulated data under different sets of parameterizations and different types of trees.

## Data Availability

Software produced in this manuscript is freely available as described from https://github.com/cnickh/wgdtree. This package includes the software for both analysis of data and simulation of data.

## Abbreviations

WGD: Whole genome duplication
SSD: Small-scale duplication
Pratio: Conditional probability ratio defined in Eq. 1
NHX: New Hampshire extended file format
MYA: Million years ago
