# Supplementary material for: WGDTree: a phylogenetic software tool to examine conditional probabilities of retention following whole genome duplication events
Source: BMC Bioinformatics. 2022 Nov 24;23:505. doi: 10.1186/s12859-022-05042-w (PMC9701042; doi:10.1186/s12859-022-05042-w)
Supplement: Supplementary file 1 — Additional file 1. This file contains 6 supplemental tables that present the analysis of simulated data under different sets of parameterizations and different types of trees. [file 12859_2022_5042_MOESM1_ESM.pdf]

Supplemental Materials

Supplemental Table 1. This table reports the accuracy (number of correctly placed events over total events) of the inference method. For each loss and small scale duplication rate combination, 1000 trees were generated. The rate unit is the event per million years. The balanced tree for this simulation placed events one speciation apart. The 95% confidence interval was generated by bootstrap resampling 1000 uniform randomly selected simulated gene trees 1000 times.

| Loss Rate | SSD Rate | Accuracy | 95% Confidence Interval |
|---|---|---|---|
| 0.01 | 0.01 | 65.7 | 65.67 - 65.83 |
| 0.01 | 0.009 | 61.67 | 61.58 - 61.76 |
| 0.01 | 0.002 | 63.32 | 63.23 - 63.42 |
| 0.01 | 0.0002 | 84.30 | 84.22 - 84.38 |
| 0.01 | 2e-05 | 90.07 | 90.00 - 90.13 |
| 0.009 | 0.01 | 89.08 | 89.01 - 89.15 |
| 0.009 | 0.009 | 60.29 | 60.20 - 60.38 |
| 0.009 | 0.002 | 63.68 | 63.59 - 63.76 |
| 0.009 | 0.0002 | 83.09 | 83.01 - 83.16 |
| 0.009 | 2e-05 | 88.33 | 88.27 - 88.40 |
| 0.002 | 0.01 | 87.12 | 87.04 - 87.19 |
| 0.002 | 0.009 | 77.76 | 77.72 - 77.79 |
| 0.002 | 0.002 | 80.44 | 80.41 - 80.48 |
| 0.002 | 0.0002 | 92.48 | 92.44 - 92.51 |
| 0.002 | 2e-05 | 96.88 | 96.86 - 96.90 |
| 0.0002 | 0.01 | 97.29 | 97.26 - 97.31 |
| 0.0002 | 0.009 | 83.82 | 83.80 - 83.84 |
| 0.0002 | 0.002 | 86.54 | 86.52 - 86.56 |
| 0.0002 | 0.0002 | 98.89 | 98.88 - 98.90 |
| 0.0002 | 2e-05 | 99.93 | 99.93 - 99.94 |
| 2e-05 | 0.01 | 99.93 | 99.93 - 99.94 |
| 2e-05 | 0.009 | 84.25 | 84.23 - 84.28 |
| 2e-05 | 0.002 | 86.88 | 86.86 - 86.90 |
| 2e-05 | 0.0002 | 98.86 | 98.85 - 98.87 |
| 2e-05 | 2e-05 | 99.97 | 99.97 - 99.97 |

Supplemental Table 2. This table reports the accuracy (number of correctly placed events over total events) of the inference method. For each loss and small scale duplication rate combination, 1000 trees were generated. The rate unit is the event per million years. The balanced tree for this simulation placed WGD events two speciation events apart. The 95% confidence interval was generated by bootstrap resampling 1000 uniform randomly selected simulated gene trees 1000 times.

| Loss Rate | SSD Rate | Accuracy | 95% Confidence Interval |
|---|---|---|---|
| 0.01 | 0.01 | 68.88 | 68.84 - 68.95 |
| 0.01 | 0.009 | 70.90 | 70.84 - 70.97 |
| 0.01 | 0.002 | 74.25 | 74.18 - 74.32 |
| 0.01 | 0.0002 | 95.00 | 94.97 - 95.04 |
| 0.01 | 2e-05 | 99.38 | 99.36 - 99.39 |
| 0.009 | 0.01 | 100.00 | 100.00 - 100.00 |
| 0.009 | 0.009 | 71.86 | 71.80 - 71.93 |
| 0.009 | 0.002 | 74.20 | 74.13 - 74.26 |
| 0.009 | 0.0002 | 94.34 | 94.30 - 94.38 |
| 0.009 | 2e-05 | 99.93 | 99.93 - 99.94 |
| 0.002 | 0.01 | 99.94 | 99.93 - 99.94 |
| 0.002 | 0.009 | 80.63 | 80.61 - 80.65 |
| 0.002 | 0.002 | 82.77 | 82.75 - 82.79 |
| 0.002 | 0.0002 | 96.46 | 96.44 - 96.49 |
| 0.002 | 2e-05 | 99.52 | 99.51 - 99.53 |
| 0.0002 | 0.01 | 99.87 | 99.87 - 99.88 |
| 0.0002 | 0.009 | 84.21 | 84.19 - 84.23 |
| 0.0002 | 0.002 | 86.27 | 86.26 - 86.29 |
| 0.0002 | 0.0002 | 98.71 | 98.70 - 98.72 |
| 0.0002 | 2e-05 | 99.90 | 99.90 - 99.90 |
| 2e-05 | 0.01 | 100.00 | 100.00 - 100.00 |
| 2e-05 | 0.009 | 84.29 | 84.28 - 84.31 |
| 2e-05 | 0.002 | 86.62 | 86.61 - 86.64 |
| 2e-05 | 0.0002 | 99.01 | 99.00 - 99.02 |
| 2e-05 | 2e-05 | 100.00 | 100.00 - 100.00 |

Supplemental Table 3. This table reports the accuracy (number of correctly placed events over total events) of the inference method. For each loss and small scale duplication rate combination, 1000 trees were generated. The rate unit is the event per million years. The catepillar tree for this simulation placed WGD events one speciation event apart. The 95% confidence interval was generated by bootstrap resampling 1000 uniform randomly selected simulated gene trees 1000 times.

| Loss Rate | SSD Rate | Accuracy | 95% Confidence Interval |
|---|---|---|---|
| 0.01 | 0.01 | 63.74 | 63.68 - 63.77 |
| 0.01 | 0.009 | 63.05 | 62.97 - 63.13 |
| 0.01 | 0.002 | 63.09 | 63.02 - 63.17 |
| 0.01 | 0.0002 | 86.45 | 86.38 - 86.52 |
| 0.01 | 2e-05 | 91.22 | 91.17 - 91.27 |
| 0.009 | 0.01 | 91.69 | 91.64 - 91.73 |
| 0.009 | 0.009 | 61.27 | 61.19 - 61.34 |
| 0.009 | 0.002 | 65.08 | 65.00 - 65.15 |
| 0.009 | 0.0002 | 84.59 | 84.53 - 84.66 |
| 0.009 | 2e-05 | 90.06 | 90.01 - 90.11 |
| 0.002 | 0.01 | 91.77 | 91.73 - 91.81 |
| 0.002 | 0.009 | 90.44 | 90.42 - 90.47 |
| 0.002 | 0.002 | 91.49 | 91.46 - 91.51 |
| 0.002 | 0.0002 | 93.80 | 93.77 - 93.83 |
| 0.002 | 2e-05 | 96.24 | 96.22 - 96.26 |
| 0.0002 | 0.01 | 95.35 | 95.33 - 95.38 |
| 0.0002 | 0.009 | 95.87 | 95.86 - 95.89 |
| 0.0002 | 0.002 | 96.27 | 96.26 - 96.29 |
| 0.0002 | 0.0002 | 99.72 | 99.71 - 99.72 |
| 0.0002 | 2e-05 | 99.93 | 99.93 - 99.94 |
| 2e-05 | 0.01 | 99.90 | 99.90 - 99.90 |
| 2e-05 | 0.009 | 96.29 | 96.29 - 96.30 |
| 2e-05 | 0.002 | 97.35 | 97.34 - 97.36 |
| 2e-05 | 0.0002 | 99.89 | 99.89 - 99.90 |
| 2e-05 | 2e-05 | 99.88 | 99.88 - 99.89 |

Supplemental Table 4. This table reports the accuracy (number of correctly placed events over total events) of the inference method. For each loss and small scale duplication rate combination, 1000 trees were generated. The rate unit is the event per million years. The catepillar tree for this simulation placed WGD events two speciation events apart. The 95% confidence interval was generated by bootstrap resampling 1000 uniform randomly selected simulated gene trees 1000 times.

| Loss Rate | SSD Rate | Accuracy | 95% Confidence Interval |
|---|---|---|---|
| 0.01 | 0.01 | 64.38 | 64.32 - 64.44 |
| 0.01 | 0.009 | 61.78 | 61.70 - 61.86 |
| 0.01 | 0.002 | 63.21 | 63.13 - 63.29 |
| 0.01 | 0.0002 | 88.52 | 88.45 - 88.59 |
| 0.01 | 2e-05 | 94.54 | 94.50 - 94.58 |
| 0.009 | 0.01 | 94.74 | 94.70 - 94.78 |
| 0.009 | 0.009 | 63.41 | 63.34 - 63.48 |
| 0.009 | 0.002 | 65.16 | 65.09 - 65.24 |
| 0.009 | 0.0002 | 85.54 | 85.48 - 85.61 |
| 0.009 | 2e-05 | 93.62 | 93.57 - 93.66 |
| 0.002 | 0.01 | 95.00 | 94.97 - 95.04 |
| 0.002 | 0.009 | 90.72 | 90.70 - 90.75 |
| 0.002 | 0.002 | 90.77 | 90.74 - 90.79 |
| 0.002 | 0.0002 | 95.49 | 95.46 - 95.51 |
| 0.002 | 2e-05 | 97.05 | 97.03 - 97.08 |
| 0.0002 | 0.01 | 97.56 | 97.54 - 97.58 |
| 0.0002 | 0.009 | 95.80 | 95.78 - 95.81 |
| 0.0002 | 0.002 | 96.29 | 96.27 - 96.30 |
| 0.0002 | 0.0002 | 99.71 | 99.70 - 99.71 |
| 0.0002 | 2e-05 | 99.90 | 99.90 - 99.90 |
| 2e-05 | 0.01 | 99.93 | 99.93 - 99.94 |
| 2e-05 | 0.009 | 94.95 | 94.80 - 94.90 |
| 2e-05 | 0.002 | 96.9 | 96.90 - 96.90 |
| 2e-05 | 0.0002 | 99.98 | 99.98 - 99.98 |
| 2e-05 | 2e-05 | 100.00 | 100.00 - 100.00 |

Supplemental Table 5. This table reports the accuracy (number of correctly placed events over total events) of the inference method. For each loss and small scale duplication rate combination, 1000 trees were generated. The rate unit is the event per million years. The catepillar tree for this simulation placed WGD events three speciation events apart. The 95% confidence interval was generated by bootstrap resampling 1000 uniform randomly selected simulated gene trees 1000 times.

| Loss Rate | SSD Rate | Accuracy | 95% Confidence Interval |
|---|---|---|---|
| 0.01 | 0.01 | 64.56 | 64.48 - 64.60 |
| 0.01 | 0.009 | 66.74 | 66.66 - 66.81 |
| 0.01 | 0.002 | 64.42 | 64.33 - 64.50 |
| 0.01 | 0.0002 | 89.36 | 89.30 - 89.42 |
| 0.01 | 2e-05 | 96.71 | 96.68 - 96.74 |
| 0.009 | 0.01 | 97.78 | 97.75 - 97.80 |
| 0.009 | 0.009 | 65.64 | 65.57 - 65.72 |
| 0.009 | 0.002 | 69.13 | 69.06 - 69.21 |
| 0.009 | 0.0002 | 86.90 | 86.84 - 86.96 |
| 0.009 | 2e-05 | 95.59 | 95.56 - 95.63 |
| 0.002 | 0.01 | 98.11 | 98.09 - 98.14 |
| 0.002 | 0.009 | 91.83 | 91.81 - 91.85 |
| 0.002 | 0.002 | 92.04 | 92.02 - 92.07 |
| 0.002 | 0.0002 | 96.38 | 96.36 - 96.41 |
| 0.002 | 2e-05 | 98.79 | 98.77 - 98.80 |
| 0.0002 | 0.01 | 98.97 | 98.96 - 98.98 |
| 0.0002 | 0.009 | 95.80 | 95.79 - 95.81 |
| 0.0002 | 0.002 | 96.67 | 96.66 - 96.68 |
| 0.0002 | 0.0002 | 99.62 | 99.61 - 99.63 |
| 0.0002 | 2e-05 | 99.90 | 99.90 - 99.91 |
| 2e-05 | 0.01 | 99.97 | 99.96 - 99.97 |
| 2e-05 | 0.009 | 96.06 | 96.05 - 96.07 |
| 2e-05 | 0.002 | 96.83 | 96.83 - 96.84 |
| 2e-05 | 0.0002 | 99.82 | 99.82 - 99.83 |
| 2e-05 | 2e-05 | 100.00 | 100.00 - 100.00 |

Supplemental Table 6. This table reports the accuracy (number of correctly placed events over total events) of the inference method. For each loss and small scale duplication rate combination, 1000 trees were generated. The rate unit is the event per million years. The catepillar tree for this simulation placed WGD events four speciation events apart. The 95% confidence interval was generated by bootstrap resampling 1000 uniform randomly selected simulated gene trees 1000 times.

| Loss Rate | SSD Rate | Accuracy | 95% Confidence Interval |
|---|---|---|---|
| 0.01 | 0.01 | 68.92 | 68.88 - 68.95 |
| 0.01 | 0.009 | 71.34 | 71.27 - 71.41 |
| 0.01 | 0.002 | 71.97 | 71.90 - 72.04 |
| 0.01 | 0.0002 | 91.73 | 91.68 - 91.78 |
| 0.01 | 2e-05 | 98.02 | 98.00 - 98.05 |
| 0.009 | 0.01 | 99.18 | 99.16 - 99.19 |
| 0.009 | 0.009 | 68.92 | 68.85 - 68.99 |
| 0.009 | 0.002 | 74.54 | 74.48 - 74.61 |
| 0.009 | 0.0002 | 91.15 | 91.10 - 91.20 |
| 0.009 | 2e-05 | 97.26 | 97.23 - 97.28 |
| 0.002 | 0.01 | 99.11 | 99.10 - 99.13 |
| 0.002 | 0.009 | 92.06 | 92.04 - 92.08 |
| 0.002 | 0.002 | 93.11 | 93.09 - 93.13 |
| 0.002 | 0.0002 | 96.66 | 96.63 - 96.69 |
| 0.002 | 2e-05 | 98.83 | 98.81 - 98.84 |
| 0.0002 | 0.01 | 99.62 | 99.61 - 99.63 |
| 0.0002 | 0.009 | 95.85 | 95.84 - 95.86 |
| 0.0002 | 0.002 | 96.32 | 96.31 - 96.33 |
| 0.0002 | 0.0002 | 99.69 | 99.68 - 99.69 |
| 0.0002 | 2e-05 | 100.00 | 100.00 - 100.00 |
| 2e-05 | 0.01 | 99.96 | 99.96 - 99.97 |
| 2e-05 | 0.009 | 96.08 | 96.07 - 96.09 |
| 2e-05 | 0.002 | 97.03 | 97.02 - 97.04 |
| 2e-05 | 0.0002 | 99.79 | 99.78 - 99.79 |
| 2e-05 | 2e-05 | 100.00 | 100.00 - 100.00 |
